# Synthesis of transition metal sulfide and reduced graphene oxide hybrids as efficient electrocatalysts for oxygen evolution reactions

**DOI:** 10.1098/rsos.180927

**Published:** 2018-09-26

**Authors:** Yu-Rim Hong, Sungwook Mhin, Jiseok Kwon, Won-Sik Han, Taeseup Song, HyukSu Han

**Affiliations:** 1 Korea Institute of Industrial Technology, 137-41 Gwahakdanji-ro, Gangneung-si, Gangwon 25440, Republic of Korea; 2 Department of Chemistry, Seoul Women's University, Seoul, Republic of Korea; 3 Korea Institute of Industrial Technology, 156 Gaetbeol-ro, Yeonsu-gu, Incheon 406-840, Republic of Korea; 4 Department of Energy Engineering, Hanyang University, Seoul 04763, Republic of Korea

**Keywords:** electrocatalyst, water splitting, oxygen evolution reaction, cobalt nickel sulfide, reduced graphene oxide, nanocomposites

## Abstract

The development of electrochemical devices for renewable energy depends to a large extent on fundamental improvements in catalysts for oxygen evolution reactions (OERs). OER activity of transition metal sulfides (TMSs) can be improved by compositing with highly conductive supports possessing a high surface-to-volume ratio, such as reduced graphene oxide (rGO). Herein we report on the relationship between synthetic conditions and the OER catalytic properties of TMSs and rGO (TMS–rGO) hybrids. Starting materials, reaction temperature and reaction time were controlled to synergistically boost the OER catalytic activity of TMS–rGO hybrids. Our results showed that (i) compared with sulfides, hydroxides are favourable as starting materials to produce the desired TMS–rGO hybrid nanostructure and (ii) high reaction temperatures and longer reaction times can increase physico-chemical interaction between TMSs and rGO supports, resulting in highly efficient OER catalytic activity.

## 1. Introduction

Electrochemical energy technologies, such as rechargeable metal–air batteries, fuel cells and water electrolysers have evoked much interest as promising alternatives for clean and sustainable energy [1–3]. The development of devices for renewable energy technologies hinges to a large extent on fundamental improvement in catalytic materials, as the oxygen evolution reaction (OER) coupled with multiple electron and proton exchange tends to be sluggish at the anodic electrode [1,4,5]. Hence, renewable energy conversion and storage devices require high-performance OER electrocatalysts to lower the activation barrier and expedite reaction kinetics [6,7]. Noble metal-based materials, such as RuO_2_ or IrO_2_, are benchmark OER catalysts; however, their high cost and low natural abundance hinder widespread commercial utilization [8]. Therefore, extensive efforts have been devoted to the design of low-cost, high-performance, abundant electrocatalysts for OER [9–18].

In recent studies, several transition metal-based compounds with boron (B), nitrogen (N), phosphorus (P) and sulfur (S), surpassed the OER activity and stability of IrO_2_ and RuO_2_ in alkaline solutions [19–23]. Among these, transition metal sulfides (TMSs) containing cobalt (Co) and nickel (Ni) have shown great promise for high-performance OER catalysts due to their desirable electrical configuration for fast charge transfer and abundant active sites for adsorption/desorption processes [24]. Material design strategies including heterometal cation doping, surface treatment and nanostructure engineering have been applied successfully to enhance the OER performance of TMSs [25–30]. Among these, hybridizing TMS nanoparticles with conductive supports is one of the most simple and cost-effective ways to boost OER catalytic performance [31–34]. Reduced graphene oxide (rGO) is a carbon (C)-based material having high surface area, robust chemical stability and high electrical conductivity, which renders it an attractive template for TMSs in electrochemical applications [19–20]. Thus, extensive research has been conducted on TMS–rGO hybrids as high-performance OER electrocatalysts. In the preparation of TMS–rGO hybrids, it is crucial that the TMS nanoparticles are well dispersed on the rGO support to achieve high electrocatalytic activity. However, *in situ* homogeneous integration of TMS nanoparticles with the rGO support is challenging due to the aggregation of rGO sheets, which hinders rapid electrolyte diffusion and reduces the surface area resulting in severe aggregation of TMS nanoparticles [35]. Close examination of the experimental variables for TMS–rGO hybrid synthesis is required to gain insight into synthesis–property relationships in TMS–rGO electrocatalysts.

Herein, we report a systematic study of synthesis–property relationships in TMS–rGO hybrids as a high-performance OER catalyst. Different starting materials were tested as precursors for TMS nanoparticles. Heat treatment conditions for *in situ* integration of the TMS nanoparticles with the rGO support were carefully optimized. Electrochemical characterizations were performed on TMS–rGO hybrids synthesized under various conditions. Based on the results, relationships between the starting materials, heat treatments and OER activity were established for TMS–rGO electrocatalysts.

## 2. Experimental section

### 2.1. Synthesis of Ni–Co hydroxide precursor

A mixture of metal acetate tetrahydrate (1.28 g) with different molar ratios of Ni/Co (2 : 1) was dissolved in 200 ml of ethanol. The solution was then refluxed at 85°C for 4 h, and the precipitate was centrifuged and rinsed with ethanol several times. The resultant powder was subsequently dried at 60°C in air.

### 2.2. Synthesis of Ni–Co sulfide precursor (NCS)

A total of 80 mg of Ni–Co hydroxide (NCO) precursors and 112.5 mg of thioacetamide were dispersed in 40 ml of ethanol solution. The mixed solution was transferred to a 200 ml Teflon^®^-lined stainless-steel autoclave and hydrothermally reacted at 120°C for 3 h. The precipitate was centrifuged and washed with ethanol several times. The resultant powder was subsequently dried at 60°C in air.

### 2.3. Synthesis of annealed NCS precursor (NCS-A)

About 100 mg of Ni–Co sulfide (NCS) powder was placed in an alumina crucible. The crucible was located at the centre of a tube furnace. Annealing treatments were performed at 350°C for 2 h under N atmosphere.

### 2.4. Synthesis of TMS–rGO hybrids

TMS–rGO hybrids were prepared using NCO, NCS and annealed NCS precursor (NCS-A) precursors via a facile one-step hydrothermal process. For a typical synthesis process, chemical exfoliation of graphite powder was performed following a modified Hummer's method to prepare graphene oxide (GO) powder. The NCO, NCS and NCS-A (40 mg) precursors were mixed with 20 ml of GO solution (concentration: 0.2 g l^–1^) and 50 ml of deionized (DI) water by stirring vigorously. Subsequent ultrasonication was performed for 20 min to homogeneously disperse the precursors in the GO solution. The mixed solution was transferred to a 200 ml Teflon^®^-lined stainless-steel autoclave and reacted hydrothermally at 200°C for 24 h, and at 120°C for 3 h. The resulting product was washed with DI water several times and freeze-dried at –50°C for 3 days.

### 2.5. Characterizations

Scanning electron microscopy (SEM; S4800, Hitachi, Tokyo, Japan) was used to study the microstructural configuration of samples. The X-ray diffraction (XRD) measurements were performed using a D/MAX-2500/PC diffractometer (Rigaku, Tokyo, Japan) with Cu-K*α* radiation (*λ* = 0.15418 nm) at 40 kV and 100 mA. The X-ray photoelectron spectroscopy (XPS) spectra were recorded using a VG ESCALAB 200i instrument (Thermo Fisher Scientific) with pass energies of 100 and 20 eV for survey and high-resolution scans, respectively. The specific surface area was estimated by N adsorption–desorption measurements (TriStar II 3020; MicroMetrics, Norcross, GA, USA) following Brunauer–Emmett–Teller (BET) theory.

### 2.6. Electrochemical characterizations

The electrochemical performance of catalysts was evaluated in 1 M KOH solution using a standard three-electrode electrochemical cell equipped with a rotating disc electrode (RDE) controlled by an electrochemistry potentiostat ion (Autolab PGSTAT; Metrohm, Herisau, Switzerland). Catalysts (4 mg) were dispersed in a Nafion^®^ solution (30 µl, 5 wt%) of water (1 ml) and ethanol (volume ratio 3 : 1) via ultrasonication. For the preparation of a working electrode, 5 µl of a homogeneous solution, formed after ultrasonication, was drop-casted onto a glassy carbon (GC) RDE (diameter: 3 mm), resulting in an approximate catalyst loading of 0.285 mg cm^−2^. The working electrode was dried overnight before electrochemical measurements. The electrolyte (1 M KOH) was purged with O_2_ before measurements, and the RDE electrode was kept rotating at 2000 r.p.m. to remove bubbles from the electrode surface. Hg/HgO and graphite rod were used as the reference and counter electrodes, respectively. The linear scanning voltammetry (LSV) was conducted at a scan rate of 5 mV s^−1^. All potentials were calibrated against a reversible hydrogen electrode (RHE), and all polarization curves were *iR*-corrected. Cyclic voltammetry (CV) was used to estimate the electrochemically active surface area (ECSA) of the catalysts. CV was performed over a potential range from 1.41 to 1.46 V versus RHE (for capacitive current flow only) to estimate the electrochemical double layer (*C*_dl_). Various scan rates (20, 40, 60, 80, 100 and 120 mV s^−1^) were tested for CV, and the difference in current density between anodic and cathodic sweeps (*ΔJ* *=* *J*_anodic_–*J*_cathodic_) at 1.435 V versus RHE was calculated, where the slope for *ΔJ* versus scan rate was equal to twice the *C*_dl_ of the catalyst. The electrochemical impedance spectroscopy (EIS) was performed in the frequency range of 0.1–100 kHz with a sinusoidal voltage (amplitude: 5 mV) to calculate charge transfer resistance (*R*_ct_).

## 3. Results and discussion

**Figure 1.**
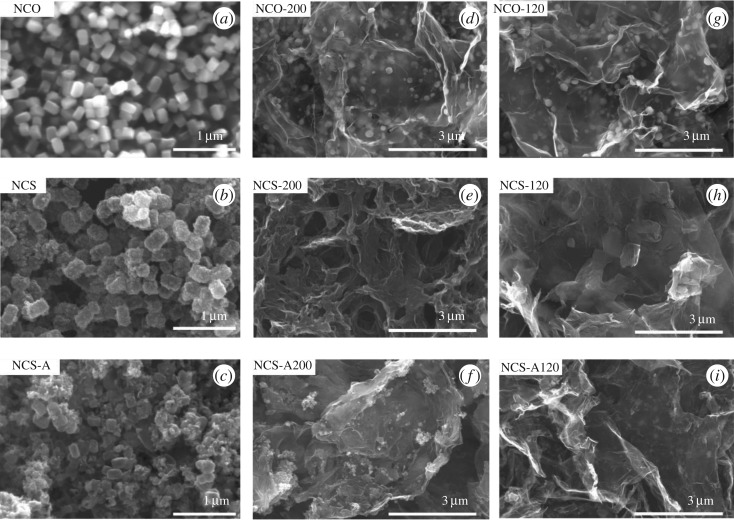
Scanning electron microscopy images for (*a–c*) NCO, NCS and NCS-A precursors, respectively, and of (*d–i*) NCO-200, NCS-200, NCS-A200, NCO-120, NCS-120 and NCS-A120 hybrids, respectively. NCO: nickel cobalt hydroxides; NCS: nickel cobalt sulfides; NCS-A post-annealed NCS with reaction temperatures of 200 or 120°C.

Three precursors, NCO, NCS and NCS-A, were used for TMS–rGO synthesis. Different reaction temperatures (200 and 120°C) and times (24 and 3 h) were tested. Figure 1 shows field-emission scanning electron microscopy (FE-SEM) images of TMS–rGO hybrids synthesized under different experimental conditions. The morphology of the TMS–rGO hybrids using NCO precursors synthesized at reaction temperatures of 200°C (NCO-200) and 120°C (NCO-120) displayed uniformly dispersed TMS nanoparticles (diameter: *ca* 100 nm) on ultrathin rGO supports. By contrast, no distinct TMS nanoparticles were observed for the hybrids using NCS precursors synthesized at reaction temperatures of 200°C (NCS-200) and 120°C (NCS-120). Also, TMS nanoparticle agglomerates were formed on the rGO supports of NCS-A200 and NCS-A120 hybrids. These results indicate that NCO precursors disperse more homogeneously in GO solution compared with NCS precursors, resulting in well-distributed TMS nanoparticles on rGO supports after the hydrothermal reaction.

**Figure 2.**
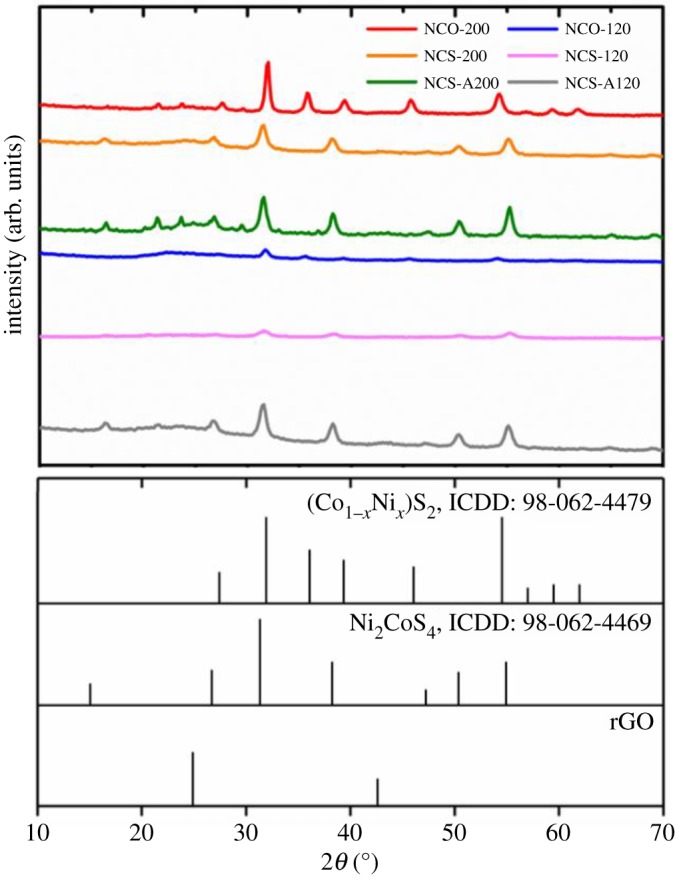
XRD patterns of NCO-200, NCS-200, NCS-A200, NCO-120, NCS-120 and NCS-A120 hybrids with the standard patterns of (Co_1−*x*_Ni_*x*_)S_2_ (ICDD: 98-062-4479), Ni_2_CoS_4_ (ICDD: 98-062-4469) and reduced graphene oxide (rGO).

XRD analysis was used to characterize the crystallographic structure of the TMS–rGO hybrids. Figure 2 shows XRD patterns of NCO-200, NCS-200, NCS-A200, NCO-120, NCS-120 and NCS-120A, respectively. The XRD patterns of NCO-200 and NCO-120 were well-matched with the reference XRD pattern of the Ni_x_Co_1−x_S_2_ phase (space group Pa-3, ICDD 98-602-4479), while NCS-200, NCS-A200, NCS-120 and NCS-A120 crystallized in the spinel Ni_2_CoS_4_ phase (space group Fd-3m, ICDD 98-062-4469). These results indicate that NCO and NCS precursors transformed into Ni_x_Co_1−x_S_2_ (space group Pa-3) and Ni_2_CoS_4_ (space group Fd-3m) phases, respectively, during the hydrothermal reaction in GO solution.

**Figure 3.**
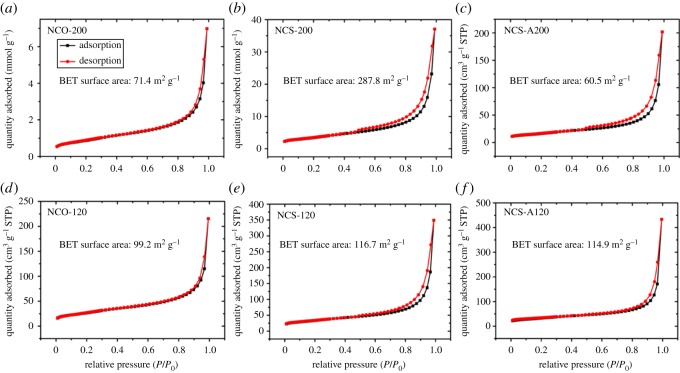
N_2_ adsorption–desorption isotherm for measuring BET surface areas of (*a*–*f*) NCO-200, NCS-200, NCS-A200, NCO-120, NCS-120 and NCS-A120, respectively. Black and red lines represent adsorption and desorption data, respectively.

Nitrogen adsorption–desorption isotherm measurements of the TMS–rGO hybrids are presented in figure 3. BET surface areas of 71.4, 287.8, 60.5, 99.2, 116.7 and 114.9 m^2^ g^−1^ were measured for NCO-200, NCS-200, NCS-A200, NCO-120, NCS-120 and NCS-A120, respectively. These values are comparable to those of other rGO-based materials reported elsewhere [36]. The higher BET surface areas for NCS-200 and NCS-120 were probably due to negligible deposition of TMS nanoparticles on rGO supports, while lower BET surface areas for NCS-A200 and NCS-A120 can be attributed to agglomeration of TMS nanoparticles as shown in figure 1.

**Figure 4.**
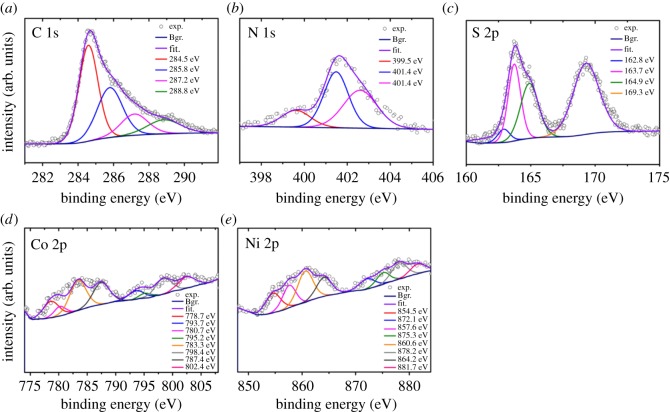
High-resolution X-ray photoelectron spectroscopy (XPS) spectra of (*a*) carbon (C 1s), (*b*) nitrogen (N 1s), (*c*) sulfur (S 2p), (*d*) cobalt (Co 2p) and (*e*) nickel (Ni 2p) for NCO-200.

**Figure 5.**
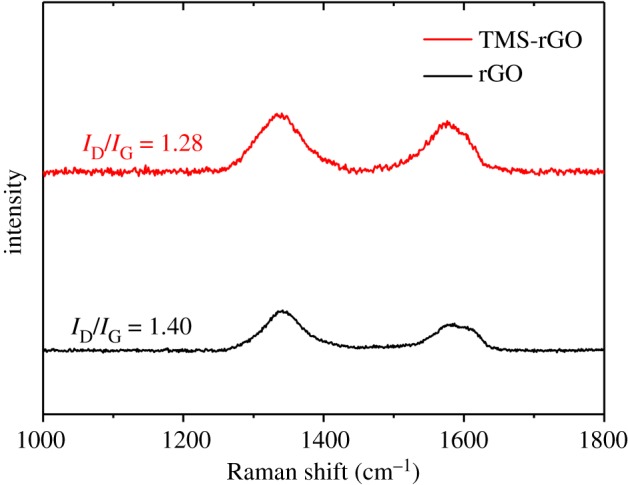
Raman spectrum of rGO and TMS–rGO.

The chemical composition and oxidation states of TMS–rGO hybrids were analysed by XPS. Representative spectra for NCO-200 are presented in figure 4. The incorporation of N into rGO was detected by high-resolution C (1s) and N (1s) spectra (figure 4*a,b*), which can be most likely originated from thioacetamide during the hydrothermal reaction. High-resolution N (1s) spectra showed that N was primarily quaternary nitrogen (401.4 and 402.5 eV) rather than pyridinic nitrogen (399.5 eV). The C (1s) spectrum was deconvoluted into four peaks with binding energies of 284.5, 285.8, 287.2 and 288.8 eV, assigned to sp^2^-hybridized C, sp^2^-C bonded to N, sp^3^-C bonded to N and C–O–C bonds, respectively [37–39]. The S (2p) spectrum contained four peaks at binding energies of 162.8 (S–Co), 163.7 (C–S–C), 164.9 (C = S) and 169.3 (C–SO_2_–) eV, indicating the formation of chemical bonds between Co, C, N and S atoms [40–42]. XPS spectra of Ni and Co showed 2p_3/2_ and 2p_1/2_ peaks for valence states of 3^+^ and 2^+^, with corresponding satellite peaks at lower energies. There were no significant differences in XPS data among the samples. The interaction between the TMS and rGO can be summarized as two things. First, the three-dimensional (3D)—a porous network of the rGO-facilitated mass transfer of the electrolyte and degassing of oxygen at the anode. The 3D porous structure of rGO can effectively anchor TMS nanoparticles on open pores. Second, Co atoms play an important role in anchoring TMS onto the rGO sheet because binding energy of the TMS and rGO was much larger for Co-anchorage (3.34 eV) than Ni-anchorage (1.03 eV) [43]. In addition, Raman spectroscopy was performed for rGO and TMS–rGO composite to study physico-chemical interaction between two components. Two distinct bands were observed at 1350 and 1590 cm^−1^ corresponding to the characteristic disorder-induced D and graphitic G bands of graphene, respectively (figure 5). The D-band is associated with disordered graphene edges, while the G band is related to the first-order scattering of the E_2 g_ mode of sp^2^-carbon domains. The peak intensity ratios between the D and the G bands (*I*_D_/*I*_G_) are often used as an indicator for the degree of defectiveness of rGO. The *I*_D_/*I*_G_ ratios were determined as 1.40 and 1.28 for rGO and rGO-TMS composite, respectively. Moreover, the D and G peaks of the TMS–rGO were downshifted from those of rGO. The downshifts of the D and G bands and the decrease in the *I*_D_/*I*_G_ ratio for the rGO–TMS can be attributed to the incorporation of Co or Ni atoms into defective sites in the rGO layer. These results reveal the interaction between the TMS and rGO.

**Figure 6.**
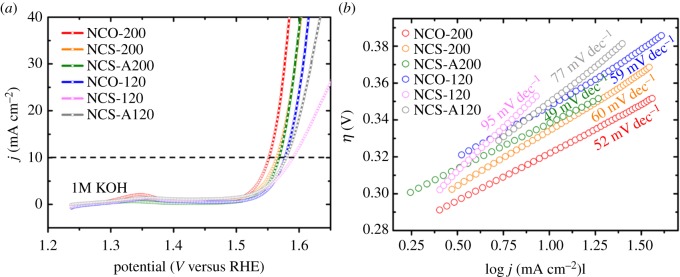
(*a*) *iR*-corrected linear sweep voltammetry (LSV) curves for NCO-200, NCS-200, NCS-A200, NCO-120, NCS-120 and NCS-A120 and (*b*) the corresponding Tafel plots measured at a scan rate of 5 mV s^−1^ in an O_2_-saturated 1.0 M KOH solution.

The electrocatalytic activities of TMS–rGO hybrids were tested using a typical three-electrode system with an RDE in a 1 M KOH aqueous solution. Linear sweep voltammetry (LSV) was conducted on all samples at a scan rate of 5 mV s^−1^. Continuous potential cycling at a scan rate of 50 mV s^−1^ was performed over a potential range from 0.95 to 1.45 V until a reproducible voltammogram was obtained. All potentials were *iR*-compensated and referenced to a hydrogen electrode (RHE). Before any electrochemical measurement, the electrolyte was bubbled with O_2_ gas for 10 min, and the RDE was rotated continuously at 2000 r.p.m. to remove gas bubbles generated on the working electrode. Polarization curves for the OER by TMS–rGO hybrids in 1.0 M KOH are shown in figure 6*a*. An anodic peak centred at about 1.35 V was observed for NCO-200 and NCO-120, associated with the reversible redox process of NCO precursors, $\text{Co}{\left(\text{OH}\right)}_{2}/\text{Ni}{\left(\text{OH}\right)}_{2}+\text{O}{\text{H}}^{-}↔\text{CoOOH}/\text{NiOOH}+{\text{H}}_{2}\text{O}+\text{e}$. NCO-200 showed the lowest overpotential (*η*) required to deliver a current density of 10 mA cm^−2^ (*η*^10^), indicating the highest OER activity of NCO-200 among the tested samples. The *η*^10^ values of NCO-200, NCS-200, NCS-A200, NCO-120, NCS-120 and NCS-A120 were as follows: 322, 335, 338, 348, 350 and 361 mV, respectively. TMS–rGO hybrids synthesized at high temperature exhibited higher OER activity than that of TMS–rGO hybrids synthesized at low temperature. These results imply that the chemical interaction between TMSs and rGO supports is crucial for enhancing the OER activity of TMS–rGO hybrids.

Tafel slope is often used for determining kinetics for electrochemical reactions. Tafel plots (*η* versus log *j*) for all samples are presented in figure 6*b*. The Tafel slopes were 52, 60, 49, 59, 95 and 77 mV dec^−1^ for NCO-200, NCS-200, NCS-A200, NCO-120, NCS-120 and NCS-A120, respectively. Smaller Tafel slopes were observed for TMS–rGO hybrids prepared at a higher temperature. Thus, faster OER reaction kinetics can be expected for TMS–rGO hybrids synthesized at a higher temperature. Tafel slopes and *η*^10^ values for all samples are summarized in table 1. For the same synthesis temperature and time, TMS–rGO hybrids using NCO precursors showed higher OER activity than that of the hybrids using NCS precursors. In addition to having a microstructural advantage (i.e. well-dispersed TMS nanoparticles on rGO supports), the presence of Co(Ni)OOH phase in NCO-200 and NCO-120 may be responsible for the enhanced OER performance [44–46].

**Table 1.** Overpotentials affording 10 mA cm^−2^, Tafel slopes, *R*_ct_ and ECSA for TMS–rGO hybrids.

materials	*η*_10_ (mV)	Tafel slope (mV dec^−1^)	*R*_ct_ (Ω)	ECSA (mF cm^−2^)
NCO-200	322	52	17.59	42.94
NCS-200	335	60	21.73	37.10
NCS-A200	338	49	17.60	25.97
NCO-120	348	59	22.26	20.24
NCS-120	350	95	202.24	1.97
NCS-A120	361	77	36.95	18.25

**Figure 7.**
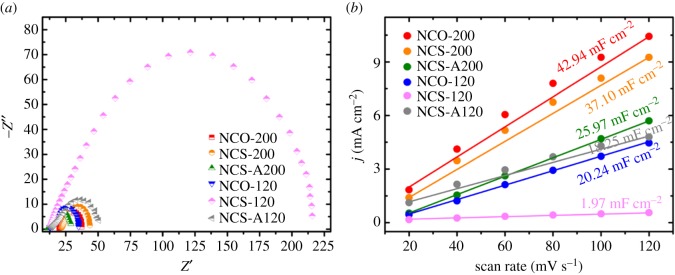
(*a*) EIS measured over a frequency range of 0.1–100 kHz under a 5 mV sinusoidal voltage and (*b*) the current density difference between anodic and cathodic sweeps as a function of scan rate for the catalysts. The slope of the fitted line was used to calculate the electrochemical ECSA.

**Figure 8.**
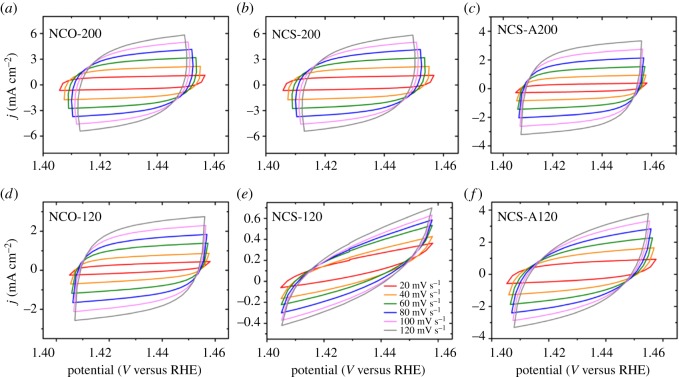
CV measurements in a voltage range of 1.4–1.46 V_RHE_ at scan rates 20, 40, 60, 80, 100 and 120 mV s^−1^ of (*a*) NCO-200, (*b*) NCS-200, (*c*) NCS-A200, (*d*) NCO-120, (*e*) NCS-120 and (*f*) NCS-A120.

EIS was performed on TMS–rGO samples and the results are shown in figure 7*a*. Charge transfer resistance (*R*_ct_) was estimated using Randall's equivalent circuit model. *R*_ct_ was calculated as 17.59, 21.73, 17.60, 22.26, 202.24 and 36.95 Ω for NCO-200, NCS-200, NCS-A200, NCO-120, NCS-120 and NCS-A120, respectively, implying that NCO-200 exhibited the fastest charge transport and reaction kinetics among the samples. The electrochemically effective surface area (ECSA) of TMS–rGO was calculated based on its double-layer capacitance (*C*_dl_), using CV at different scan rates. CV cyclings at different scan rates were performed over the voltage range of 1.41–1.45 V to obtain the capacitive currents related only to double-layer charging (figure 8). The difference in current density between anodic and cathodic sweeps (*ΔJ* *=* *J*_anodic_–*J*_cathodic_) at 1.435 V is plotted as a function of the scan rate, in which the slope is equal to twice that of *C*_dl_ [40,47]. Figure 7*b* shows a linear plot with slope values of 42.94, 37.10, 25.97, 20.24, 1.97 and 18.25 mF cm^−2^ for NCO-200, NCS-200, NCS-A200, NCO-120, NCS-120 and NCS-A120, respectively. Based on this, the high OER activity of NCO-200 is also attributable to its large ECSA. *R*_ct_ and ECSA for the samples are summarized in table 1.

Mass activity and turnover frequency (TOF) are often used to define quantitatively the OER activity of catalysts. Mass activity can be calculated as

**Figure 9.**
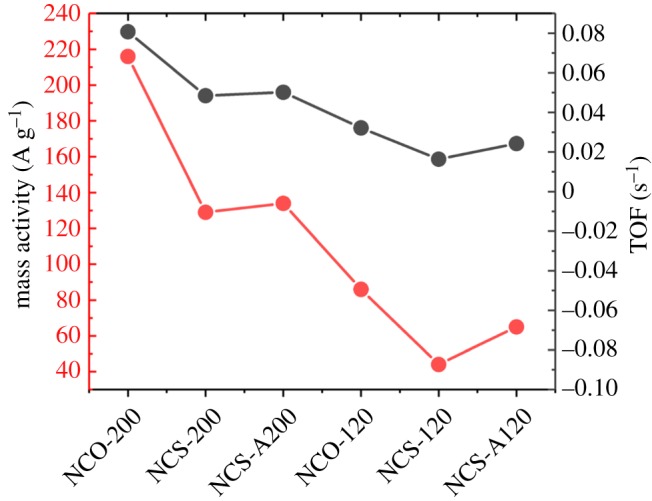
Mass activity (red line) and turnover frequencies (TOFs) (black line) at *η* = 360 mV for NCO-200, NCS-200, NCS-A200, NCO-120, NCS-120 and NCS-A120.

**Figure 10.**
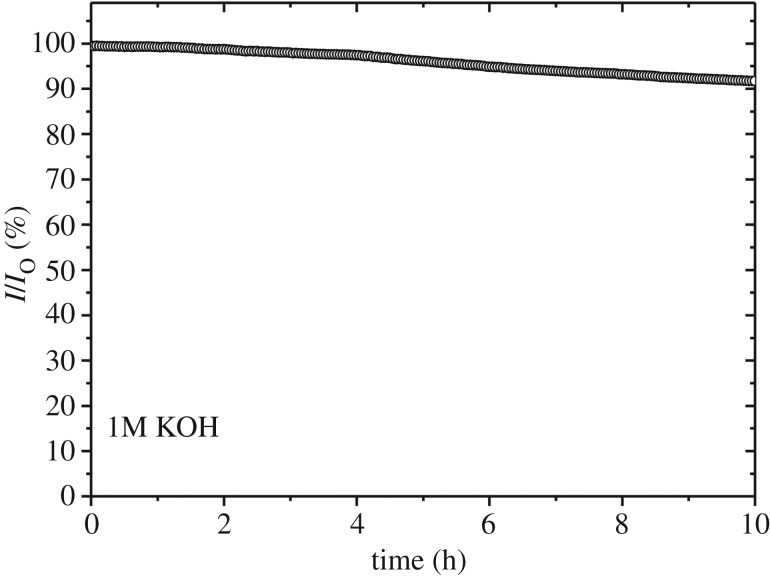
Chronoamperometry curve of NCO-200 under the applied voltage of 1.6 V_RHE_ in 1 M KOH solution.

3.1 $$\text{mass activity }=\frac{\;j}{c},$$

where *j* is the current density at a specific potential and *c* is the loading amount of catalyst on the electrode, which is 0.285 mg cm^−2^ in this work. TOF is calculated as follows:

3.2 $$\text{TOF}=\frac{\;j\;\times A}{4\times F\times n},$$

where *A* is the area of the electrode, *F* is the Faraday constant (96 485 C mol^−1^) and *n* is the number of moles of the active metal sites (i.e. Co or Ni) in the catalysts, as determined by quantitative XPS analyses. According to quantitative XPS analysis for the representative sample of NCO-200, C and O compose about 97 at% (mainly C) and hence the rest (approx. 3 at%) consists of TMS. Assume that most of GO is reduced, then weight percentage for rGO and TMS will be about 75.9 and 24.1 wt%, respectively. Note that since there will be a different number of oxygen groups attached to individual rGO sheets and degree of reduction is also different within each rGO sheet, it is quite difficult to determine the exact molecular weight for rGO. As we determined weight percentage of TMS in the composite, approximately 0.0068 mg of TMS is present in the working electrode (24.1 wt% of the loading amount of 0.285 mg cm^−2^). Thus, a total number of mol (*n*) for Co and Ni in NCO-200 will be about 5.58 × 10^−8^ mols. Figure 9 shows the mass activities of the catalysts at an overpotential of 360 mV. The mass activity of NCO-200 was highest among the samples and reached about 216 A g^−1^ at the given potential. This value is about fivefold higher than that of NCS-120 and twofold higher than that of NCS-200. The TOFs for the catalysts, as shown in figure 9, are as follows: 0.08 s^−1^ (NCO-200), 0.04 s^−1^ (NCS-200), 0.05 s^−1^ (NCS-A200), 0.03 s^−1^ (NCO-120), 0.01 s^−1^ (NCS-120) and 0.02 s^−1^ (NCS-A120). The TOF of NCO-200 was about eightfold higher than that of NCS-120 and twofold higher than that of NCS-200. These results indicate that TMS–rGO hybrids synthesized at higher temperature have better OER catalytic activity, regardless of precursor type. Among the hybrids synthesized at the same heat treatment conditions, the NCO precursor provided better OER performance to TMS–rGO hybrids compared with NCS and NCS-A precursors. Long-term stability is also important for practical applications of TMS–rGO, since surface oxidation is thermodynamically unavoidable for TMS under alkaline OER conditions. The electrocatalytic stability of NCO-200 was tested by chronoamperometric measurements at an overpotential of 340 mV. After 10 h of OER, the current density of NCO-200 decreased by less than 10% of its initial value (figure 10), demonstrating a good stability in aqueous alkaline media. Thus, electrochemical characterizations showed that high-temperature hydrothermal processes using NCO as precursors are preferred for synthesizing TMS–rGO hybrids with high-performance OER activity. Electrocatalytic OER activity of TMS–rGO is compared with the recently reported state-of-the-art electrocatalysts (table 2), demonstrating the excellent performance of TMS–rGO for water oxidation.

**Table 2.** Comparison of OER catalytic activity of TMS–rGO with reported state of the art OER electrocatalysts on glassy carbon (GC) electrode in alkaline media.

catalysts	electrolyte	*η*^10^ (mV)	Tafel slope (mV dec^−1^)	references
TMS–rGO (NCO-200)	1 M KOH	322	52	this study
^48^Co-CoO/N-rGO	0.1 M KOH	∼400	68	[48]
^49^Co9S8@MoS2/CNFs	1 M KOH	430	61	[49]
^50^NG-CNT	0.1 M KOH	520	141	[50]
*^51^NS/rGO-Co*	1 M KOH	265	72	[51]
^52^NiCo_2_S_4_ NA/CC	1 M KOH	340	89	[52]
^53^CuCo_2_S_4_ nanosheets	0.1 M KOH	337	n.a.	[53]
^54^CoFe hydroxysulfides/graphene	0.1 M KOH	358	79	[54]

**Figure 11.**
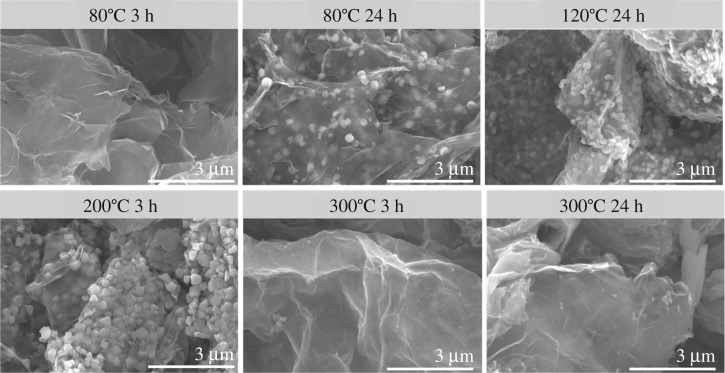
FE-SEM images of TMS–rGO hybrids synthesis at different temperature and time.

**Figure 12.**
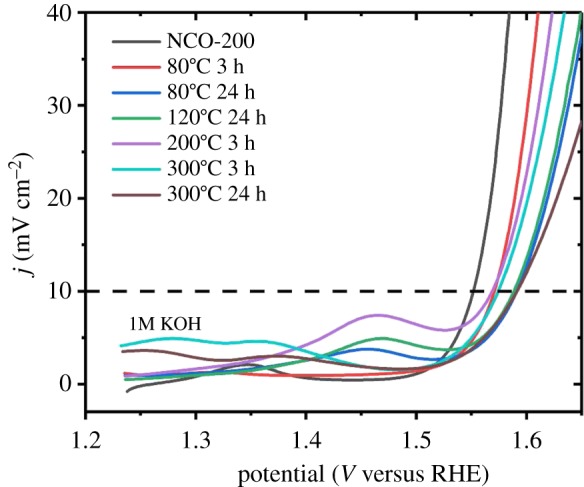
LSV plots for TMS–rGO hybrids synthesis at different temperature and time.

To further investigate synthesis–property relationships in TMS–rGO hybrids, more parallel experiments were performed at different temperature, time and pressure. For investigating the effect of temperature and time, the reactions were carried out at 80°C for 24 and 3 h, 120°C for 24 h, 200°C for 3 h, and 300°C for 24 and 3 h, and the morphologies and electrocatalytic activities of the resulting samples were analysed by FE-SEM and LSV, respectively. NCO was used as a precursor for all control experiments due to its higher electrocatalytic activity than NCS. Based on SEM images, TMS–rGO hybrid was not formed when the reaction temperature was 80°C and the reaction time was 3 h (figure 11). However, for longer reaction time up to 24 h, TMS–rGO hybrid was successfully synthesized at 80°C, indicating that a lower limit for reaction temperature is around 80°C. Importantly, TMS–rGO hybrids were formed at reaction temperatures of 120 and 200°C for both reaction times of 3 and 24 h. Hence, the optimal reaction temperature for the synthesis of TMS–rGO should be around 120–200°C. Short reaction time (3 h) at 200°C results in high aggregation of TMS particles, and thus longer reaction time may be favoured for the synthesis of TMS–rGO hybrid at a reaction temperature between 120 and 200°C. In addition, a high reaction temperature of 300°C was not able to synthesize TMS–rGO hybrids regardless of reaction time, implying that upper limit for reaction temperature is in between 200 and 300°C. Furthermore, more experiments were performed to study how pressure affect the reaction. The pressure in the 200 ml Teflon^®^-lined stainless-steel autoclave was about 10 bar at 200°C. Since we cannot precisely control the pressure inside the autoclave used in this work (lack of pressure controller), autoclaves with different volume and size of Teflon^®^-lined stainless-steel autoclave, 50 and 100 ml, resulting in built-in pressure as 30 and 20 bar at 200°C, were tested, respectively. The synthesis was reproduced using the different autoclaves. As a control experiment, the synthesis was also performed at atmospheric pressure while reaction temperature and time were kept as same. The morphologies of the resulting samples were analysed by FE-SEM, which is not shown here. Higher pressure over 10 bar results in significant agglomeration of TMS particles as well as large grain growth of the particles, while lower pressure results in no anchorage of TMS particles onto rGO sheets. Hence, optimal pressure for the synthesis of TMS–rGO hybrid should be around 10 bar. Lastly, electrocatalytic activity of TMS–rGO hybrids synthesized at different temperature and time was analysed by LSV in 1.0 M KOH solution (figure 12). TMS–rGO synthesized at 200°C for 24 h using NCO as precursor exhibits the lowest overpotential for affording current density of 10 mA cm^−2^. In conclusion, optimal synthesis conditions for TMS–rGO hybrid should be controlled at a reaction temperature of 120–200°C, reaction time of 3–24 h, and pressure of 10 bar in order to obtain excellent OER catalytic activity in alkaline solution.

## 4. Conclusion

In summary, we studied the synthesis–property relationships in TMS–rGO hybrids as efficient OER electrocatalysts. Three precursors were used for the synthesis: NCO, NCS and NCS-A. Different reaction temperatures, times and pressures were tested. Structural analysis confirmed that using the NCO precursor confers an advantage in synthesizing TMS–rGO hybrids in which TMS nanoparticles are dispersed homogeneously on rGO supports. In addition, high reaction temperatures (approx. 200°C), longer reaction times (approx. 24 h) and pressure of approximately 10 bar are preferable to enhance the OER electrocatalytic activity of TMS–rGO hybrids, possibly due to the improved physico-chemical interaction between TMS nanoparticles and rGO supports. The TMS–rGO hybrid synthesized using NCO precursors at 200°C for 24 h exhibited the highest OER activity among the catalysts; specifically, an overpotential of 322 mV, 10 mA cm^−2^ current density, Tafel slope of 52 mV dec^−1^, mass activity of 220 A g^−1^ and TOF of 0.08 s^−2^ were achieved.

## Data accessibility

There are no additional data to accompany this manuscript. All relevant datasets are within the main body of the manuscript. All protocols and software used are stated fully in the methodology section. There are no coding lists for this research.
